# Systemic activation of NLRP3 inflammasome and plasma α-synuclein levels are correlated with motor severity and progression in Parkinson’s disease

**DOI:** 10.1186/s12974-019-1670-6

**Published:** 2020-01-08

**Authors:** Zheng Fan, Yu-Ting Pan, Zhi-Yuan Zhang, Hui Yang, Shu-Yue Yu, Yan Zheng, Jing-Hong Ma, Xiao-Min Wang

**Affiliations:** 1 Department of Pharmacology, School of Basic Medical Sciences, Capital Medical University, Beijing, China; 2 Department of Neurology, Xuanwu Hospital of Capital Medical University, No.45 Changchun Street, Beijing, 100053 China; 3 Department of Neurobiology, School of Basic Medical Sciences, Capital Medical University, No.10 Xitoutiao, Youanmenwai, Beijing, 100069 China; 4 Department of Physiology, School of Basic Medical Sciences, Capital Medical University, Beijing, China; 5 Core Facility Center, Capital Medical University, Beijing, China; 6 Preventive Medicine, School of Public Health, Capital Medical University, Beijing, China

**Keywords:** NLRP3 inflammasome, Interleukin-1β, α-Synuclein, Parkinson’s disease, Inflammation

## Abstract

**Background:**

Emerging evidence indicates that inflammasome-induced inflammation plays a crucial role in the pathogenesis of Parkinson’s disease (PD). Several proteins including α-synuclein trigger the activation of NLRP3 inflammasome. However, few studies examined whether inflammasomes are activated in the periphery of PD patients and their possible value in the diagnosis or tracking of the progress of PD. The aim of this study was to determine the association between inflammasome-induced inflammation and clinical features in PD.

**Methods:**

There were a total of 67 participants, including 43 patients with PD and 24 controls, in the study. Participants received a complete evaluation of motor and non-motor symptoms, including Hoehn and Yahr (H-Y) staging scale. Blood samples were collected from all participants. The protein and mRNA expression levels of inflammasomes subtypes and components in peripheral blood mononuclear cells (PBMCs) were determined using western blotting and RT-qPCR. We applied Meso Scale Discovery (MSD) immunoassay to measure the plasma levels of IL-1β and α-synuclein.

**Results:**

We observed increased gene expression of NLRP3, ASC, and caspase-1 in PBMCs, and increased protein levels of NLRP3, caspase-1, and IL-1β in PD patients. Plasma levels of IL-1β were significantly higher in patients with PD compared with controls and have a positive correlation with H-Y stage and UPDRS part III scores. Furthermore, plasma α-synuclein levels were also increased in PD patients and have a positive correlation with both UPDRS part III scores and plasma IL-1β levels.

**Conclusions:**

Our data demonstrated that the NLRP3 inflammasome is activated in the PBMCs from PD patients. The related inflammatory cytokine IL-1β and total α-synuclein in plasma were increased in PD patients than controls, and both of them presented a positive correlation with motor severity in patients with PD. Furthermore, plasma α-synuclein levels have a positive correlation with IL-1β levels in PD patients. All these findings suggested that the NLRP3 inflammasome activation-related cytokine IL-1β and α-synuclein could serve as non-invasive biomarkers to monitor the severity and progression of PD in regard to motor function.

## Background

Parkinson’s disease (PD) is one of the most common neurodegenerative disorders and is characterized by selective and progressive loss of dopaminergic neurons projecting from the substantia nigra pars compacta (SNc) to the corpus striatum, leading to an extrapyramidal motor disorder with bradykinesia, resting tremor, rigidity, and postural instability. The loss of dopamine is linked to the presence of Lewy bodies in the SNc and other brain regions, and the abnormal intracellular inclusions which contain aggregated α-synuclein are the classical pathological hallmark of PD. Nigrostriatal dopaminergic neurodegeneration correlates with the Parkinsonian motor features, but involvement of other peripheral and central nervous system regions leads to a wide range of non-motor features, for example, hyposmia, rapid eye movement sleep behavior disorder, depression, and constipation [1]. The diagnosis of PD is currently dependent on the presence of motor deficits, while the pathology stage of Lewy body deposition has already advanced, with more than 50% of dopaminergic neurons have been lost when motor symptoms are evident [2]. The availability of objective fluid biomarkers specifically associated with motor or non-motor features of PD could allow reliable prediction of clinical outcomes.

Although the etiology of PD is not clear, it seems likely that inflammation plays a role in the pathogenesis of the disease. Indeed, microglia activation in the SNc and other affected regions has been detected in postmortem brains of PD patients, as well as increased levels of pro-inflammatory cytokines [3, 4]. In addition, systemic inflammation has also been suggested to contribute to neurodegeneration in PD, as lymphocyte infiltration has been observed in the brains of PD patients and in animal models of PD [5, 6]. The source of these pro-inflammatory cytokines in the brain is therefore primarily microglia and other infiltrating peripheral myeloid cells. High levels of pro-inflammatory cytokines, including tumor necrosis factor (TNF), interleukin (IL)-1β, and IL-6, are critical signaling molecules of immune activation and expressed in the brains, cerebrospinal fluid (CSF), and serum of patients with PD [7–9]. Although a number of studies showed associations between inflammatory cytokines and PD, those associations were inconsistent for individual cytokines and between studies [7–16].

Inflammasomes are a group of cytosolic multiprotein protein complexes that represent a major innate immune response platform, which recognize a large number of stimuli, such as danger-associated molecular patterns (DAMPs) and pathogen-associated molecular patterns (PAMPs). Once the inflammasome sensor molecules are activated by a trigger, they undergo conformational changes leading to the loss of an autoinhibited state, thus undergoing oligomerization, in which they can trigger the helical fibrillar assembly of a downstream adaptor protein called apoptosis-associated speck-like protein (ASC). Subsequently, ASC induces monomeric pro-caspase-1 aggregation to initiate pro-caspase-1 self-cleavage to become the active caspase-1, which results in the maturation and secretion of pro-inflammatory cytokines IL-1β and IL-18, as well as pyroptotic cell death [17]. Neuroinflammatory cascades rely on the activation of inflammasome, which has been proved crucial in PD [18, 19]. In our previous studies, we had reported the activation of NLRP3 inflammasome involved in the pathogenesis of PD and might be a potential target for PD therapy [20, 21]. But the connection between the clinical features of PD and the inflammasome-mediated inflammatory response has not been clearly defined.

Although several studies have assessed the possible increases in pro-inflammatory cytokines in plasma or serum of PD patients, their possible value in the diagnosis or tracking of the progress of PD is not clear. Therefore, the present study determined whether inflammasomes are activated in peripheral blood mononuclear cells (PBMCs) from PD patients. Furthermore, we evaluated the correlation between the related inflammatory cytokine IL-1β and clinical symptoms of PD, as well as the plasma levels of α-synuclein. In this way, we aimed to assess their relationship with the disease severity and progression of PD.

## Methods

### Subjects

We used the UK Parkinson’s Disease Society Brain Bank criteria [22] for PD diagnosis to recruit 43 patients with PD consecutively from the specialized neurodegenerative outpatient clinic in the Departments of Neurology, Beijing Xuanwu Hospital, Capital Medical University, from October 2017 to April 2018. Data collection included demographic information such as sex, age, and disease duration.

Control participants were recruited from the local community. A total of 24 age-matched controls were selected based on the following criteria: (1) no PD or secondary Parkinsonism; (2) no inflammatory, infectious, or autoimmune diseases in peripheral and central systems; and (3) no obvious cognitive impairment or psychiatric symptoms. In addition, individuals who had used corticosteroids, anti-inflammatories, or antibiotics in the 4 weeks prior to the study were excluded.

### Evaluation of motor and non-motor symptoms

Detailed clinical information was obtained from the patient’s history and neurological examination. Parkinsonism was diagnosed by movement disorders specialists experienced in Parkinsonian disorders. Motor symptom severity was evaluated using the motor subscale of the Unified Parkinson’s Disease Rating Scale (UPDRS part III) [23]. Non-motor symptoms of patients with PD were assessed by the Unified Parkinson’s Disease Rating Scale (UPDRS part I). The UPDRS scores were obtained in the “on” state of the disease. The modified Hoehn and Yahr (H-Y) staging scale was used to establish the stage of PD [24].

### Plasma and blood mononuclear cell isolation

Within 2–4 h after blood donation, up to 10 ml anti-coagulated blood was separated in centrifuge tubes (BD Biosciences, MD, USA) prefilled with 5 ml Ficoll Paque (GE Healthcare, Uppsala, Sweden), according to the instructions of the manufacturer. After being centrifuged at 3500 rpm for 10 min at 4 °C, plasma supernatant was harvested and stored at − 80 °C until further analysis. The enriched cell fraction containing PBMCs was harvested and washed twice in 10 ml phosphate-buffered saline (PBS). Cell pellets were shock-frozen for cryopreservation at − 80 °C until further usage.

### RNA isolation and RT-qPCR analysis

Total RNA was extracted from cryopreserved PBMC pellets using RNeasy mini Kit (Qiagen, Hilden, Germany) according to the manufacturer’s instructions. The RNA samples were treated with 10 μL RNase-free DNase to remove residual genomic DNA. The RNA quantity and purity was determined using NanoPhotometer spectrophotometer at 260 nm/280 nm (Implen, CA, USA). Only RNA without DNA contamination and degradation of 26S rRNA was used for subsequent cDNA synthesis. For expression analysis, 0.2 μg of the total RNA was reverse transcribed using FastQuant RT Kit (Tiangen BioTech, Beijing, China). For real-time PCR, Power SYBR Green master mix (Life Technologies) was added to appropriate cDNA samples and specific primers in QuantStudio 5 Real-Time PCR cycler. Primers for the quantitation real-time PCR were as follows: NLRP3, 5′-AAGGGCCATGGACTATTTCC-3′ (forward) and 5′-GACTCCACCCGATGACAGTT-3′ (reverse); NLRP1, 5′-CAGGCAGCACAGATCAACAT-3′ (forward) and 5′-GTGACCTTGAGGACGGAGAA-3′ (reverse); NLRC4, 5′-TAGCCGAGCCCTTATTCAAA-3′ (forward) and 5′-ACCTTCTCGCAGCAAATGAT-3′ (reverse); ASC, 5′-AAGCCAGGCCTGCACTTTAT-3′ (forward) and 5′-CTGGTACTGCTCATCCGTCA-3′ (reverse); Caspase-1, 5′-CCGAAGGTGATCATCATCCA-3′ (forward) and 5′-ATAGCATCATCCTCAAACTCTTCTG-3′ (reverse); IL-1β, 5′-CTGAAAGCTCTCCACCTCCA-3′ (forward) and 5′-CCAAGGCCACAGGTATTTTG-3′ (reverse); GAPDH, 5′-GAAGGTGAAGGTCGGAGTC-3′ (forward) and 5′-GAAGATGGTGATGGGATTTC-3′ (reverse). The amplification program was performed using the following cycling conditions: initial denaturation for 2 min at 95 °C, followed by 40 cycles of 95 °C for 15 s and 60 °C for 1 min. Quantitative analysis of gene expression was performed with a StepOne Plus Real Time PCR System (Life technologies). Relative expression was determined from cycle thresholds (CT) by using individual standard amplification curves relative to the corresponding mean expression of reference transcript (GAPDH). Fold changes in the expression of genes of interest were calculated using the ΔΔCt method.

### Western blotting

PBMC samples were lysed in RIPA buffer and homogenized by ultrasound. Cell protein lysates were quantified by Bradford assays (Bio-Rad, Hercules, CA, USA). Forty micrograms of proteins were electrophoresed through 8–15% SDS-polyacrylamide gel and blotted to PVDF membrane. Blots were probed with the following primary antibodies: anti-NLRP3 (1:1000, Cell Signaling Technology, Beverly, MA, USA), anti-caspase-1 (1:1000, Adipogen Corporation, CA, USA), anti-IL-1β (1:800, R&D Systems, Minneapolis, USA), and anti-GAPDH (1:5000, Sigma-Aldrich, MO, USA). Immunoreactive proteins were detected using HRP-conjugated secondary antibodies (Santa Cruz Biotechnology, USA) and Pierce ECL Western blotting substrate (Thermo Scientific, Waltham, MA, USA). The membranes were scanned and analyzed in a Chemiluminescence Imaging System (ProteinSimple, San Jose, CA, USA). For relative quantification, signals of equal exposure times were densitometrically analyzed with ImageJ and normalized to corresponding GAPDH signals.

### Measurement of plasma IL-1β and α-synuclein concentration by Meso Scale Discovery (MSD) immunoassay

V-PLEX human IL-1β kits (K151QPD) and U-PLEX human α-synuclein kits (K151WKK) were purchased from Meso Scale Discovery (Rockville, MD, USA), and the protocol was followed as recommended. Briefly, sample or 7-point diluted calibrators were added in antibody-coated 96-well plates, and plates were sealed and incubated at room temperature for 2 h with shaking. Plates were aspirated and washed three times with PBST (PBS + 0.05% Tween-20). Twenty-five microliters of Sulfo-Tag secondary antibody in diluent was then added, and the plate was incubated for 2 h with shaking. Plates were washed three times in PBST as above, and 150 μL of 2× reading buffer was added. Plates were read immediately in MESO QuickPlex SQ 120.

### Statistical analysis

All data were analyzed by GraphPad Prism 7.0 software (GraphPad Software Inc., La Jolla, CA). All values are expressed as the mean ± SEM. Due to unequal group sizes and non-normal distribution of some variables, non-parametric tests were used for group comparisons and correlation analyses. ANOVA was used for the analysis of relative gene expressions between PD patients and controls. The variations between all groups were examined by Mann-Whitney tests or Student’s *t* tests. Discrete variable comparisons were performed using Fisher’s exact test. The relationships between study parameters were examined via Spearman’s correlation test. For each test, *p* values < 0.05 were considered statistically significant.

## Results

### The characteristics of participants

A total of 43 patients and 24 healthy people (control group) were included in the study. As shown in Table 1, there were no statistical differences between groups according to age and sex (*p* = 0.695, *p* = 0.549, respectively).

**Table 1:** Clinical characteristics of study participants

Variables	PD patients (*n* = 43)	Healthy control (*n* = 24)	*p* value
Gender (female/male)	19/24	11/13	0.549^a^
Age (years)	58.40 ± 1.37	57.92 ± 1.58	0.695^b^
Disease duration (years)	2.27 ± 0.34	N.A.	N.A.
H-Y	1.93 ± 0.11	N.A.	N.A.
UPDRS part I	8.72 ± 0.76	N.A.	N.A.
UPDRS part III	31.35 ± 2.05	N.A.	N.A.

Numbers are expressed as mean ± SEM

*N.A*. not available, *PD* Parkinson’s disease, *H-Y* Hoehn-Yahr staging scale, *UPDRS* Unified Parkinson’s Disease Rating Scale

^a^Fisher’s exact test

^b^Mann-Whitney test

### The NLRP3 inflammasome is activated in PBMCs from PD patients

**Fig. 1:**
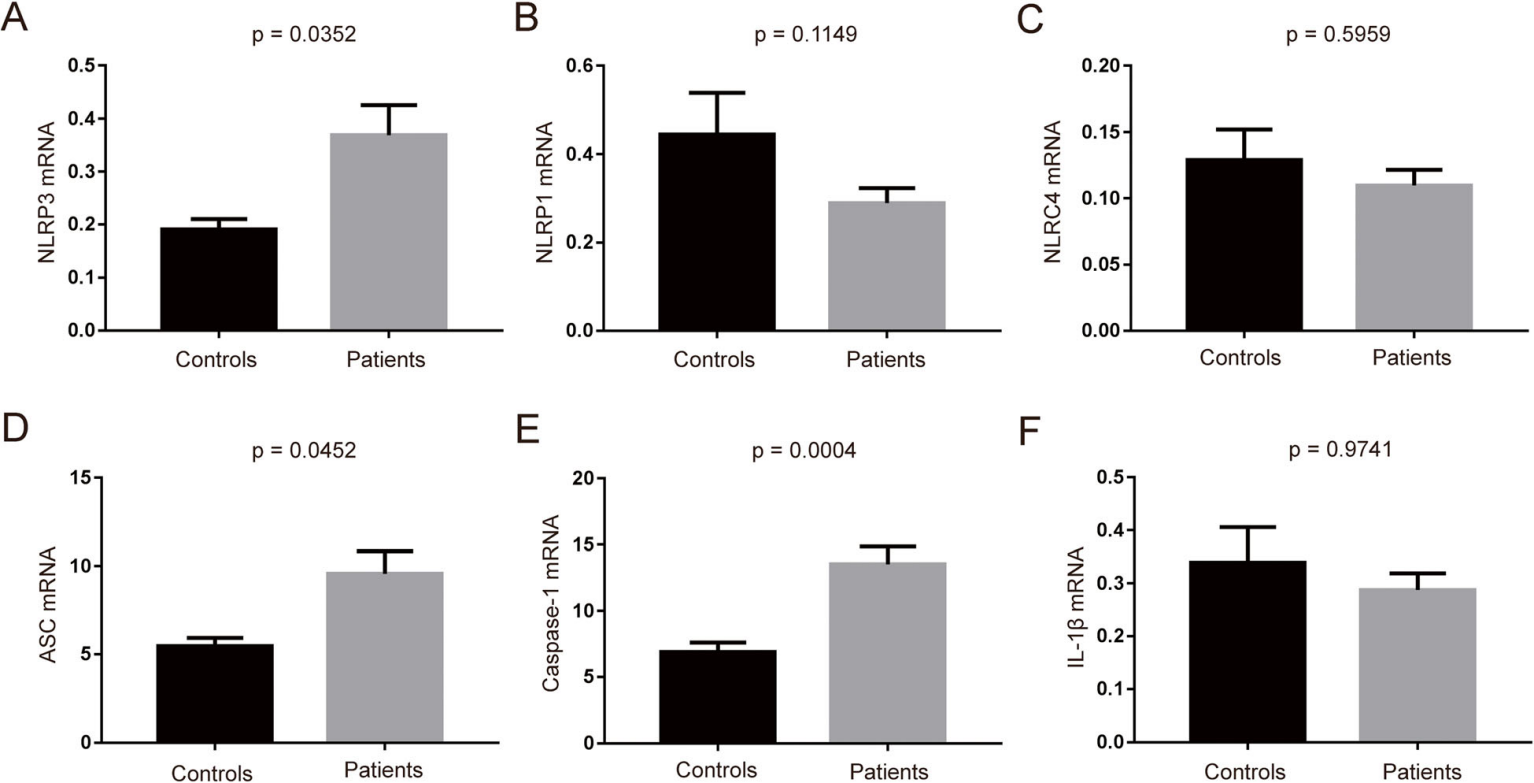
Expression of inflammasome-related genes in the PBMCs of PD patients and healthy controls. **a**–**c** NLRP3, NLRP1, and NLRC4 mRNA expression levels relative to GAPDH were determined by RT-qPCR analysis as described in the “Methods” section. **d**–**f** ASC, Caspase-1, and IL-1β mRNA expression relative to GAPDH were determined by RT-qPCR analysis. *n* = 24 for control, *n* = 43 for PD groups respectively. Data are expressed as mean ± SEM, one-way ANOVA

The focus of this study is on the NLRP1, NLRP3, and NLRC4 inflammasomes because they have garnered the most attention in the progression of neurodegenerative diseases [25]. Quantitative analysis of gene expression was performed by real-time PCR, and relative expression was determined by using individual standard amplification curves of each transcript relative to the corresponding mean expression of GAPDH. As shown in Fig. 1, NLRP3 gene expression was higher in the PD patients compared to the control group (Fig. 1a, *p* = 0.0352), while NLRP1 and NLRC4 gene expression did not differ between the groups (Fig. 1b, c). The gene expression of downstream adaptor protein ASC was also higher in the PD patients (Fig. 1d, *p* = 0.0452). To examine, whether the increased gene expression of inflammasome components resulted in increased activation of the inflammasome multiprotein complex, we next determined the gene expression of its effector proteins, caspase-1 and IL-1β. The results showed caspase-1 gene expression was significantly higher in the PD patients (Fig. 1e, *p* = 0.0004), while IL-1β gene expression was not statistically significant between the groups (Fig. 1f).

**Fig. 2:**
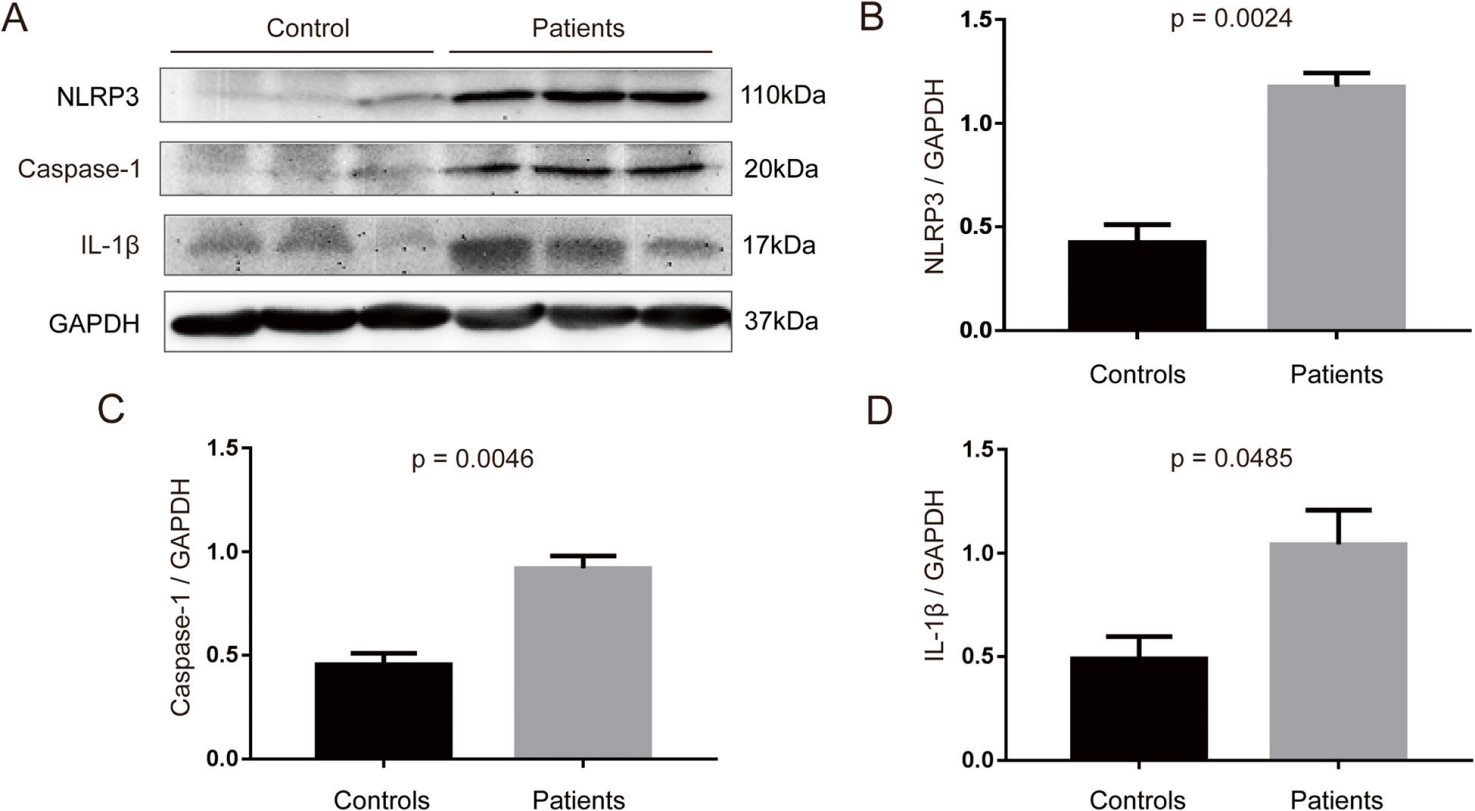
Expression of NLRP3 inflammasome activation-related proteins in the PBMCs of PD patients and healthy controls. **a** NLRP3, Caspase-1, and IL-1β protein levels were analyzed by Western blotting using PBMCs from three representative patients, compared with three healthy age- and sex-matched control subjects. **b**–**d** Protein expression levels were quantified by densitometric analysis (IOD, integrated optical intensity) of three different Western blotting and normalized to GADPH signal. *n* = 3 for each group. Data are expressed as mean ± SEM, one-way ANOVA

Then, we sought to determine whether the protein levels of those components of inflammasome had changed. Western blotting of PBMC lysates showed increased NLRP3 (*p* = 0.0024), active caspase-1 (*p* = 0.0046), and mature IL-1β (*p* = 0.0385) protein expression relative to GAPDH in PBMCs from PD patients compared with controls suggesting NLRP3 inflammasome activation in periphery (Fig. 2a–d).

### Plasma IL-1β levels increased in PD patients and have a positive correlation with H-Y stage and UPDRS part III scores

**Fig. 3:**
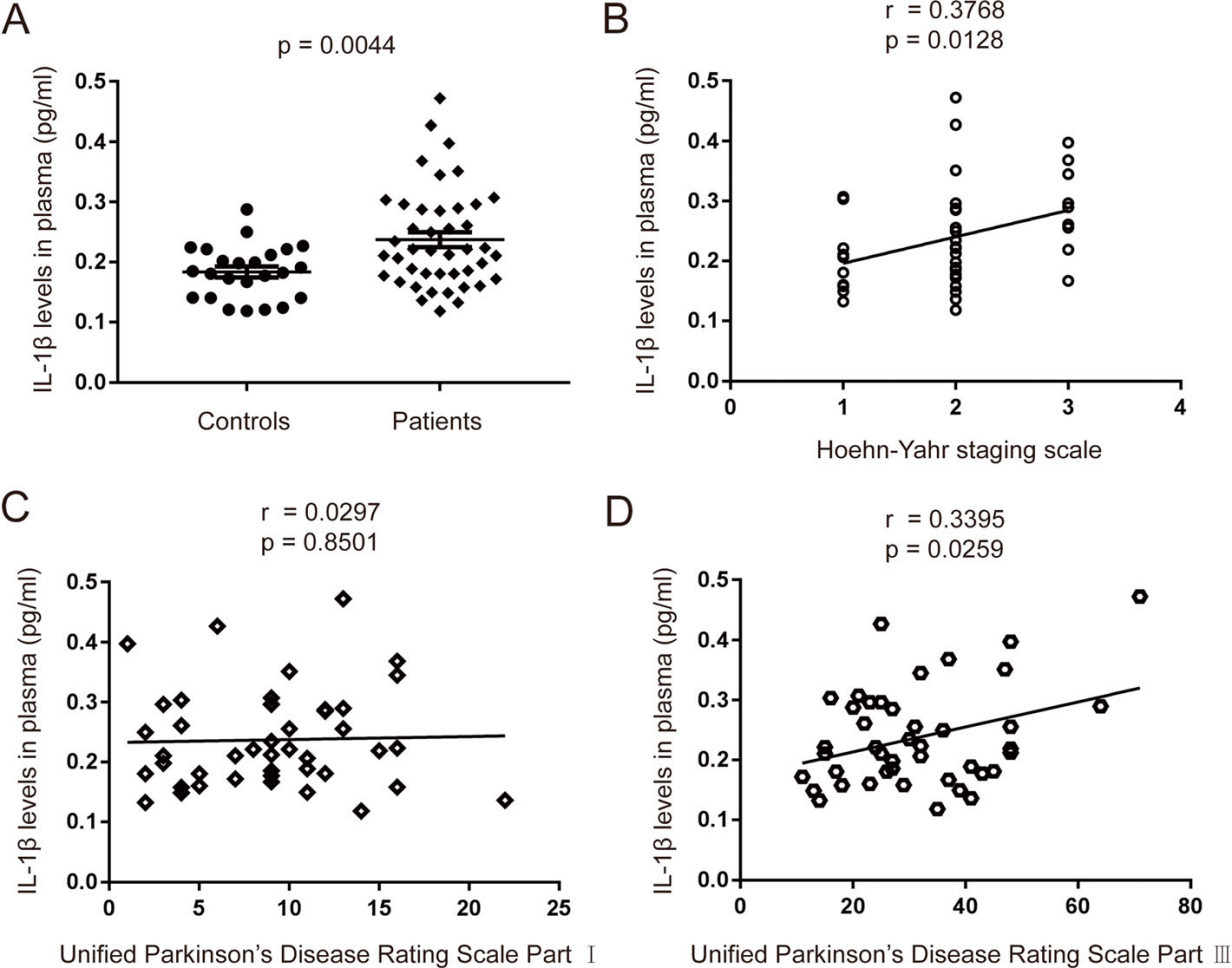
Plasma levels of IL-1β in PD patients and healthy controls, and the correlation with clinical characteristics. **a** Measurement of IL-1β concentration by MSD immunoassay in plasma from PD (solid diamond) and healthy control (solid circles) subjects. *n* = 24 for control, *n* = 43 for PD groups respectively. Data are expressed as mean ± SEM, one-way ANOVA. **b** Correlation of IL-1β levels in plasma from PD patients with H-Y stage. **c** Correlation of IL-1β levels in plasma from PD patients with UPDRS part I scores. **d** Correlation of IL-1β levels in plasma from PD patients with UPDRS part III scores. The correlation was established by calculating correlation coefficients

Plasma is the most commonly analyzed sample type, being relatively easy to obtain and offering the prospect of readily measurable biomarkers of immunological or inflammatory diseases. In this study, we used MSD immunoassay which has the higher sensitivity to detect IL-1β concentrations (detection range 0.04–375 pg/ml) in plasma from PD patients and the controls. As shown in Fig. 3a, plasma IL-1β levels of the PD patients (0.2373 ± 0.0126 pg/ml) were significantly higher than those of the controls (0.1835 ± 0.009 pg/ml, *p* = 0.0044). Furthermore, the plasma levels of IL-1β showed a positive correlation with the H-Y stage scale of PD patients (Fig. 3b, *r* = 0.3768, *p* = 0.0128). We next examined whether the plasma levels of IL-1β correlated with disease severity, either in terms of motor or non-motor symptoms. For the non-motor symptom severity evaluation, we observed that plasma IL-1β levels had no correlation with the UPDRS part I scores (Fig. 3c, *r* = 0.0297, *p* = 0.8501). However, a positive correlation was found between motor symptom severity (as assessed by UPDRS part III scores) and plasma levels of IL-1β (Fig. 3d, *r* = 0.3395, *p* = 0.0259). This data suggests that IL-1β has a role in the pathogenesis of PD and correlated with motor severity and progression in PD.

### Plasma α-synuclein levels increased in PD patients and have a positive correlation with UPDRS part III scores

**Fig. 4:**
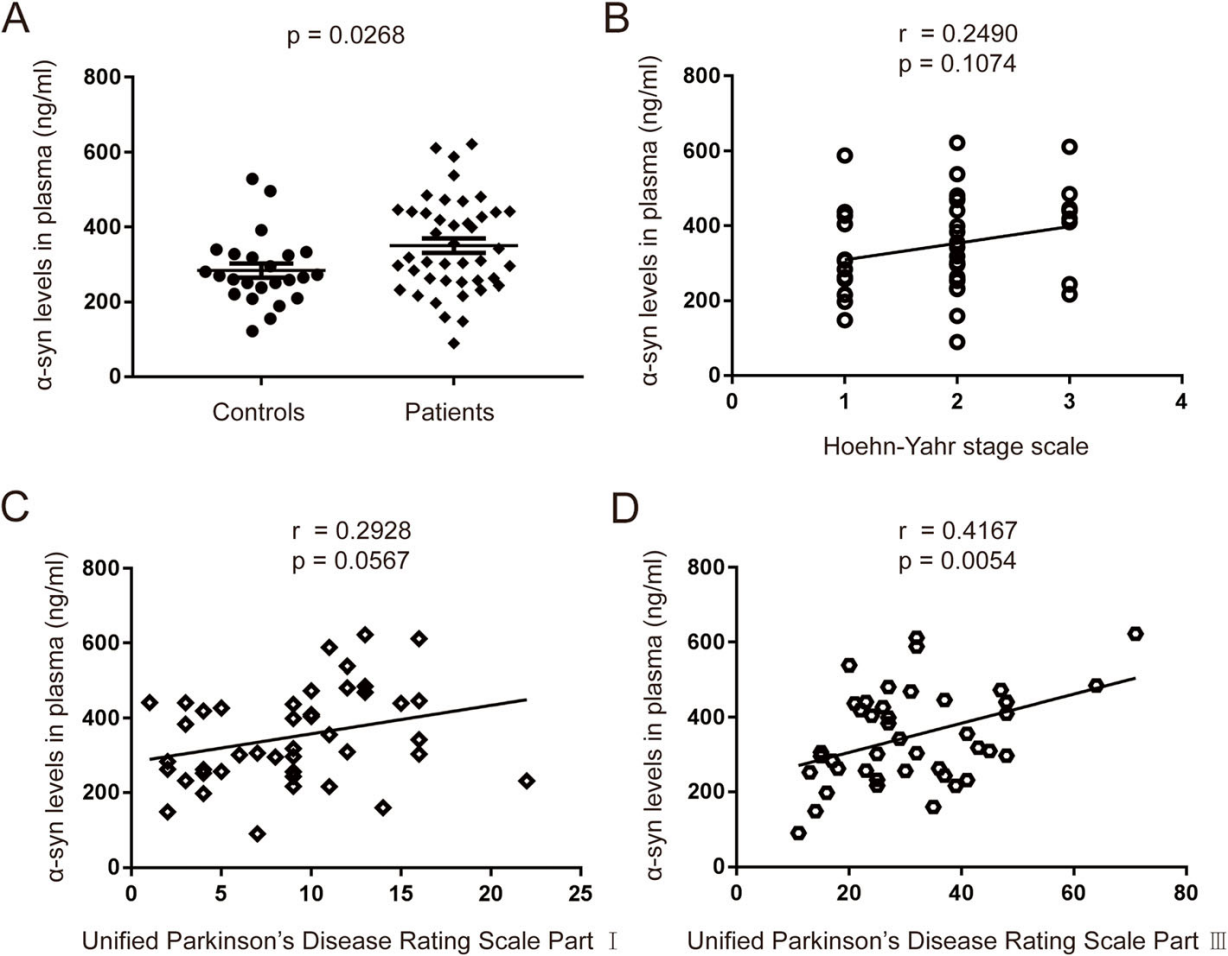
Plasma levels of α-synuclein in PD patients and healthy controls and the correlation with clinical characteristics. **a** Measurement of α-synuclein concentration by MSD immunoassay in plasma from PD (solid diamond) and normal control (solid circles) subjects. *n* = 24 for control, *n* = 43 for PD groups respectively. Data are expressed as mean ± SEM, one-way ANOVA. **b** Correlation of α-synuclein levels in plasma from PD patients with H-Y stage. **c** Correlation of α-synuclein levels in plasma from PD patients with UPDRS part I scores. **d** Correlation of α-synuclein levels in plasma from PD patients with UPDRS part III scores. The correlation was established by calculating correlation coefficients

It has been previously reported that there is a microglial uptake of misfolded α-synuclein and subsequent activation of the NLRP3 inflammasome [20]. So we examined the plasma levels of α-synuclein in PD patients and the controls. As shown in Fig. 4a, plasma levels of α-synuclein were higher in PD patients (350.2 ± 19.20 ng/ml) compared with health controls (283.6 ± 19.03 ng/ml; *p* = 0.0268). Furthermore, the plasma levels of α-synuclein had no correlation with the H-Y stage scale of PD patients (Fig. 4b, *r* = 0.2490, *p* = 0.1074), and weak correlation with UPDRS part I scores but was not statistically significant (Fig. 4c, *r* = 0.2928, *p* = 0.0567). However, a significant positive correlation was found between UPDRS part III scores and plasma levels of α-synuclein (Fig. 4d, *r* = 0.4167, *p* = 0.0054). The results suggested that a higher plasma α-synuclein level was associated with motor symptom severity of PD.

### Plasma α-synuclein levels have a positive correlation with IL-1β levels in PD patients

**Fig. 5:**
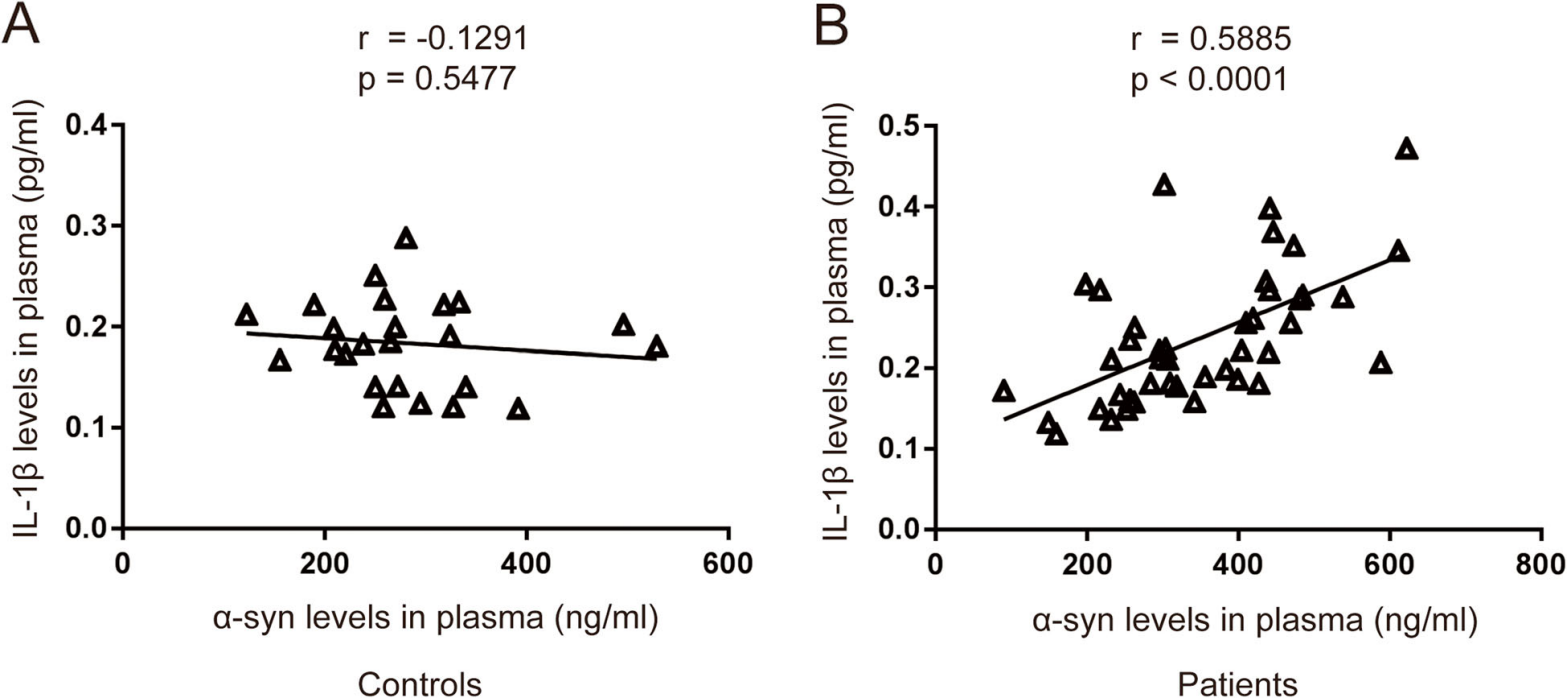
The correlation between α-synuclein levels with IL-1β levels in PD patients and healthy controls. **a** Correlation of α-synuclein levels with IL-1β levels in plasma from healthy controls. **b** Correlation of α-synuclein levels with IL-1β levels in plasma from PD patients. The correlation was established by calculating correlation coefficients

We next examined whether the plasma levels of α-synuclein correlated with IL-1β levels. We observed that plasma α-synuclein levels showed high positive correlation with IL-1β of plasma in PD patients (Fig. 5b, *r* = 0.5885, *p* < 0.0001), while no correlation was found between the plasma levels of IL-1β and α-synuclein in healthy controls (Fig. 5a, *r* = − 0.1291, *p* = 0.5477). The results suggested that a higher plasma α-synuclein level was associated with NLRP3 inflammasome-induced inflammation in the pathogenesis of PD.

## Discussion

As obtained from the analysis of PBMCs and plasma of patients with PD, the present study provides compelling evidence for the systemic activation of the NLRP3 inflammasome in this disorder. Compared to healthy individuals, patients’ PBMCs were found to display increased levels of NLRP3 inflammasome-related mRNA transcripts and protein expression. Also, we found a significant increase in the plasma levels of IL-1β and α-synuclein in PD patients. Correlation analyses revealed a positive correlation between plasma IL-1β levels and the H-Y stage, as well as motor symptom severity in PD patients. We demonstrated that plasma levels of α-synuclein were positively correlated with motor symptom severity and IL-1β levels in patients with PD, which is the pro-inflammatory cytokine of NLRP3 inflammasome activation.

Elevated levels of inflammatory cytokines in the brain, peripheral organs, CSF, and serum of PD patients support the existence of functional interconnections between the immune and nervous systems. Several reports showed that dopamine neuronal loss in PD originates from neuroinflammation and is triggered by systemic circulating inflammatory molecules [26, 27]. Within innate immunity, inflammasomes act as an important immune defense in central and peripheral tissues. Inflammasome complexes generally have three main components: a cytosolic pattern-recognition receptor (PRR), the enzyme caspase-1, and an adaptor protein that facilitates the interaction between the two [28]. NOD-like receptor (NLR) is one type of PRRs that can be activated by many endogenous or exogenous activators. Three subtypes, NLRP1, NLRP3, and NLRC4, are the most widely studied inflammasomes within the central nervous system [29]. In this study, we found the mRNA expression of NLRP3, but not NLRP1 and NLRC4, was increased in the PBMCs from PD patients compared with the controls. However, the mRNA expression of IL-1β was not upregulated in PD patients, while its protein expression was increased in PD patients detected by Western blotting. A recent study showed that several microRNAs can target mRNA transcripts of genes coding for components of inflammasome complexes [30]. One possible explanation is that the microRNA-mediated post-transcriptional control regulates IL-1β expression.

The easy access to blood samples has led to a number of studies analyzing peripheral inflammatory cytokine levels in patients with PD compared with healthy control individuals in the hope of better understanding the etiology of PD and providing candidate biomarkers for the disease. Inflammatory markers, such as interleukins, are critical signaling molecules of immune activation that exert effects in the brain and in the periphery. The production and the maturation of IL-1β, which is important in many inflammatory processes, were controlled by the activation of NLRP3 inflammasome. Although a number of studies showed associations between IL-1β concentrations and PD, those results were inconsistent between studies [9, 12, 31]. The reason might be that some cytokines are present at very low abundance (< 1 pg/ml) in peripheral blood including IL-1β, and as such, they are near the limit of detection of current assays. This makes it difficult to detect small changes in their abundance that may accompany a disease state. Thus, we used MSD immunoassay which has higher sensitivity to detect IL-1β concentrations in plasma. In this study, we found a significant elevation of plasma levels for IL-1β in patients with PD compared to healthy control subjects. Importantly, our data indicated that the plasma levels of IL-1β were found to positively correlate with the H-Y stage scale and motor symptom severity (as assessed by UPDRS part III scores), but had no significant correlation with non-motor symptom severity (as assessed by UPDRS part I scores). In this study, non-motor symptoms were evaluated using UPDRS part I, which is the section of the UPDRS designed to evaluate non-motor symptoms. The scale includes 13 items, each one evaluating the severity of non-motor symptoms relevant in PD. However, some studies preferred to use the Non-Motor Symptom Assessment Scale (NMSS) to assess non-motor symptoms, which is a measure for 30 non-motor symptoms that are recognized as relevant in PD due to their prevalence and burden [32]. According to a recent study, there is a close relation between corresponding components and total scores of both scales [33]. Therefore, both scales are valid and available for use, although they differ in structure and contents. Our observations supported the hypothesis that peripheral inflammation may progress with disease progression, and the levels of plasma IL-1β were associated with the disease severity of PD patients.

α-Synuclein holds promise as a biomarker because it is a major component of Lewy bodies and can be found in peripheral tissues and body fluids. The meta-analysis revealed the concentration of total α-synuclein in plasma of PD patients was higher compared to that of controls [34]. Consistent with these observations, our results also demonstrated higher plasma α-synuclein levels in PD patients compared with controls. Furthermore, we observed that plasma α-synuclein levels were positively correlated with motor severity, while had weak correlation with non-motor symptoms but was not statistically significant. Previous studies reported that α-synuclein deposition has been associated with widespread Lewy body-like pathology and concomitant motor impairment [35]. A positive correlation was observed between motor symptoms scores and α-synuclein levels in plasma of PD patients, suggesting the severity of neurodegeneration and burden of α-synuclein pathological conditions are closely coupled during disease progression in PD. Both the brain and the blood tissues are natural sources of α-synuclein, as such, it is currently unknown whether defining the concentration of α-synuclein in plasma reflects the actual levels of the brain-resident α-synuclein. Further studies are warranted to elucidate the mechanism involved in the transport of α-synuclein from the central nervous system to the peripheral blood and the pathophysiology behind the clinical symptoms of PD. Our study also showed that plasma α-synuclein levels were associated with the concentration of IL-1β in patients with PD. It had been demonstrated that insoluble α-synuclein fibrils induced monocytes to release IL-1β following the activation of NLRP3 inflammasome inducing a strong inflammatory response in PD [36].

The major advantage of this study was studying the relation of inflammasome-induced inflammation in the periphery and clinical features of PD. Another advantage of this study is the use of the MSD immunoassay to detect plasma levels of IL-1β and α-synuclein in control subjects and PD patients, which manifests a low interference and high specificity for detecting plasma target proteins compared to the traditional ELISA method. However, our study has some limitations. First, the number of normal controls and patients with PD was not comparable, and the relatively small number of enrolled subjects and the cross-sectional design of this study may limit the extent to which our data can be extrapolated to all patients with PD. Future, large cohort studies with a long follow-up period are needed to validate our results. Second, in this study, we had not found a significant correlation between plasma α-synuclein levels and non-motor symptoms. One possible explanation is that we only assessed non-motor symptoms using UPDRS part I, which is the section of the UPDRS designed to evaluate non-motor symptoms. Detailed tests evaluating individual non-motor symptoms are warranted for further assessment of the correlation between plasma α-synuclein levels and non-motor symptoms in PD patients. Third, contributing to the conflicting results for serum or plasma quantities of total α-synuclein in patients with PD compared to controls [32, 37], future studies concomitantly incorporating assessments of total and phosphorylated α-synuclein in plasma may be needed to better predict PD progression.

Elevated levels of numerous pro-inflammatory cytokines were detected within the cortex, basal ganglia, and/or limbic regions on postmortem exam of PD brains [38]. However, the exact mechanisms by which cytokine levels are elevated in the peripheral blood of PD patients remain controversial. It is believed that neuroinflammation in the central nervous system of PD may induce a systemic inflammatory response to activate mononuclear cells in the peripheral blood to express and produce more cytokines during the disease development and progression of PD. We hope that this study will provide valuable information for the association between peripheral NLRP3-induced inflammation and PD. After validation of these target proteins as diagnosis or disease progression biomarkers in PD, the next step is to target the biomarkers for potential new and better treatments for the growing number of individuals with PD worldwide.

## Conclusions

In summary, our data indicate that the NLRP3 inflammasome is activated in the PBMCs from PD patients as compared with the health controls. The related inflammatory cytokine IL-1β and total α-synuclein in plasma were increased in PD patients than controls, and both of them presented a positive correlation with motor severity in patients with PD. Furthermore, plasma α-synuclein levels have a positive correlation with IL-1β levels in PD patients. Our findings suggest that the NLRP3 inflammasome activation-related cytokine IL-1β and α-synuclein could serve as non-invasive biomarkers to monitor the severity and progression of PD in regard to motor function.

## Data Availability

The datasets and materials supporting the conclusions of this article are included within the article.

## Abbreviations

ASC: Apoptosis-associated speck-like protein
CSF: Cerebral spinal fluid
DAMPs: Danger-associated molecular patterns
H-Y: Hoehn and Yahr staging scale
IL-1β: Interleukin-1β
MSD: Meso Scale Discovery
NLR: NOD-like receptor
NMSS: Non-Motor Symptom Assessment Scale
PAMPs: Pathogen-associated molecular patterns
PBMCs: Peripheral blood mononuclear cells
PD: Parkinson’s disease
PRR: Pattern-recognition receptor
SNc: Substantia nigra pars compacta
TNF: Tumor necrosis factor
UPDRS: Unified Parkinson’s Disease Rating Scale
